# Repeated Exposure to Illusory Sense of Body Ownership and Agency Over a Moving Virtual Body Improves Executive Functioning and Increases Prefrontal Cortex Activity in the Elderly

**DOI:** 10.3389/fnhum.2021.674326

**Published:** 2021-05-31

**Authors:** Dalila Burin, Ryuta Kawashima

**Affiliations:** ^1^Department of Advanced Brain Science, Institute of Development, Aging and Cancer, Tohoku University, Sendai, Japan; ^2^Smart Aging International Research Center, Tohoku University, Sendai, Japan

**Keywords:** immersive virtual reality, sense of body ownership, sense of agency, executive functions, Stroop task, functional near-infrared spectroscopy, dorsolateral prefrontal cortex, virtual intervention

## Abstract

We previously showed that the illusory sense of ownership and agency over a moving body in immersive virtual reality (displayed in a first-person perspective) can trigger subjective and physiological reactions on the real subject’s body and, therefore, an acute improvement of cognitive functions after a single session of high-intensity intermittent exercise performed exclusively by one’s own virtual body, similar to what happens when we actually do physical activity. As well as confirming previous results, here, we aimed at finding in the elderly an increased improvement after a longer virtual training with similar characteristics. Forty-two healthy older subjects (28 females, average age = 71.71 years) completed a parallel-group randomized controlled trial (RCT; UMIN000039843, umin.ac.jp) including an adapted version of the virtual training previously used: while sitting, participants observed the virtual body in a first-person perspective (1PP) or a third-person perspective (3PP) performing 20 min of virtual high-intensity intermittent exercise (vHIE; the avatar switched between fast and slow walking every 2 min). This was repeated twice a week for 6 weeks. During the vHIE, we measured the heart rate and administered questionnaires to evaluate illusory body ownership and agency. Before the beginning of the intervention, immediately after the first session of vHIE, and at the end of the entire intervention, we evaluated the cognitive performance at the Stroop task with online recording of the hemodynamic activity over the left dorsolateral prefrontal cortex. While we confirm previous results regarding the virtual illusion and its physiological effects, we did not find significant cognitive or neural improvement immediately after the first vHIE session. As a novelty, in the 1PP group only, we detected a significant decrease in the response time of the Stroop task in the post-intervention assessment compared to its baseline; coherently, we found an increased activation on left dorsolateral prefrontal cortex (lDLPFC) after the entire intervention. While the current results strengthen the impact of the virtual full-body illusion and its physiological consequences on the elderly as well, they might have stronger and more established body representations. Perhaps, a longer and increased exposure to those illusions is necessary to initiate the cascade of events that culminates to an improved cognitive performance.

## Introduction

The relationship between the sense of body ownership (i.e., the conscious subjective feeling of owning one’s own body) (Gallagher, 2000) and the sense of agency (i.e., the experience of controlling one’s motor acts and, through them, the external events) (Haggard, 2017) is intricate. Despite several theories and experimental efforts having been attempted over time, they can be organized into three main factions: (1) those who support the “additive model,” where agency entails the sense of body ownership, so they are strongly connected, but the sense of agency includes additional components (Tsakiris et al., 2006; Longo and Haggard, 2009; Kalckert and Ehrsson, 2014; Ma and Hommel, 2015; Pia et al., 2016); (2) those who, in contrast, support the “independence model,” where body ownership and agency are separate experiences with different neural basis (Farrer and Frith, 2002; Schwartz et al., 2005; Tsakiris et al., 2010); and (3) the more recent supporters of the “interactive model,” where body ownership and agency are partially connected at the level of sensory-related signals and shared neural network, but they can be treated as separate experiences at the level of additional specific processes (Pyasik et al., 2018; Seghezzi et al., 2019). Even comparing studies that involve the same type of measurement, there are frequently controversial results (see, for example, Tsakiris et al., 2010 and Seghezzi et al., 2019 for brain imaging data or Kalckert and Ehrsson, 2012 and Dummer et al., 2009 for behavioral data).

However, in most daily life activities, we do not perceive any discrepancies or mismatches. On the other hand, experimental situations have demonstrated how these two components can be deconstructed (also independently of each other) and reconstructed over an entity different from the actual own body by exploiting the same multisensory integration process that leads to the assimilation of the minimal self (Tsakiris, 2010): in the rubber hand illusion (RHI) (Botvinick and Cohen, 1998), the sense of body ownership is deconstructed (Moseley et al., 2008, 2012; Burin et al., 2017; Pfister et al., 2020) and reconstructed over a prosthetic hand that is simultaneously touched (and seen) with the real subject’s hand (not seen) by integrating body-related afferent signals (Pyasik et al., 2018, 2019).

Despite the RHI procedure having been revised in several different ways (Ehrsson, 2007; Kalckert and Ehrsson, 2012; Burin et al., 2017), it has been outdated by its full-body version in immersive virtual reality (IVR), where the entire body can be displayed and the illusion can be triggered by the sole visual stimulation (Maselli and Slater, 2013; Kokkinara et al., 2016; Slater, 2018; Burin et al., 2019a): through an IVR visor, the virtual body (also called avatar) can be entirely displayed and such environment allows the control of several variables (somatic features of the avatar, spatial location, movement control, etc.) (Kilteni et al., 2012; Banakou et al., 2013, 2016; Peck et al., 2013). Crucially, the virtual body can be shown in a first-person perspective, being spatially coincident with the real one, overlapping it (if the person wearing the visor looks down to where his/her body is supposed to be, he/she sees the virtual body) and immediately creating the illusion of ownership, without the necessity of additional stimulations (Kokkinara et al., 2016; Burin et al., 2019a; Neyret et al., 2020). Different with respect to the RHI, the avatar in IVR can replicate in real-time complex movements through a tracking system (Banakou and Slater, 2014). However, it can also reproduce animated movements (such as walking or running) (Kokkinara et al., 2016), which can be attributed to one’s motor intention, possibly thanks to *a posteriori* reconstruction of the sense of agency (meaning “this virtual body is mine–the virtual body is moving–those movements are mine”) (Burin et al., 2019a).

Recent studies have shown that the illusory feeling of ownership and agency over the virtual body creates the necessary conditions to induce effects on the physiological (Martini et al., 2013; Kokkinara et al., 2016; Fossataro et al., 2020) or even components higher than the mere perceptual level, such as social (Peck et al., 2013; Banakou et al., 2018), neural (Seinfeld et al., 2021), or cognitive functions: concerning the latter, in our previous study, we demonstrated on young healthy participants acute improvement of cognitive (executive) functions after a high-intensity intermittent exercise performed exclusively by the considered-as-own virtual body (Burin et al., 2019c, 2020). We argued that, despite the participants being completely still, the feeling of ownership and agency over the virtual body (only if displayed in a first-person perspective) (Kokkinara et al., 2016) induced a cascade of events (from the physiological activation of the heart rate to the increased neural activity over task-related areas), culminating in the improved cognitive performance immediately after the virtual exercise, comparable to what happens after a similar training performed by one’s own physical body (Hyodo et al., 2016; Kujach et al., 2017). These results have potential clinical applications, such as improvement of bodily and cognitive functions for those who cannot perform physical activity.

While the beneficial effects of a physical (or virtual, as in this case) training seem to be more defined in young participants, it is still quite unclear whether the same phenomenon can be observed in the elderly as well for several reasons: from the perspective of multisensory illusions, such as the RHI, the elderly can experience it, subjectively and objectively, but they report different levels of strength of the illusion, probably because of an altered sense of body ownership (Kuehn et al., 2018; Zeller and Hullin, 2018; Riemer et al., 2019). Also, from the behavioral and cortical activation points of view, the differences between young and old people are not entirely clear: a very recent study described that, after mild-intensity physical activity, the young as well as the elderly showed an acute (30 min after the training) improvement of overall inhibitory functions, specifically at the Stroop task, even though they might show some differences (Fujihara et al., 2021). Clearly, whether the same training is also effective if performed virtually (as with young participants) is unknown. Lastly, despite the elderly representing the typical control group for neurological patients (most of them are, in fact, elderly), it is quite complicated for them to perform this kind of high-intensity exercise (it might, for example, enhance the risk of falls).

Consequently, questions remain open: does this virtual exercise benefit a different population, such as the elderly? Are there differences between the acute and long-term impacts of this virtual exercise?

In the present study, in order to answer these questions, we adapted the same virtual training (Burin et al., 2019c, 2020) to test its efficacy on a sample of 42 physically and neurologically healthy elderly (over 60 years old) and to compare the acute and long-term impacts on cognitive, physiological, and neural functions. We conducted a parallel-group randomized controlled trial (RCT) composed of a 6-week (twice a week) IVR intervention, each session including 20 min of virtual high-intensity intermittent exercise (vHIE), alternating the avatar between fast walking and slowly walking every 2 min: while the participants were sitting still, they observed the virtual body, either in a first-person perspective (1PP, the experimental group) or a third-person perspective (3PP, the control group), performing the virtual exercise. We measured the heart rate and administered questionnaires during the virtual training to evaluate the presence of the full-body illusion on a physiological and subjective level. We assessed cognitive performance with the Stroop task [with the online recording of hemodynamic activity over the left dorsolateral prefrontal cortex (lDLPFC) with a functional near-infrared spectroscopy (fNIRS) device] at three time points: before the beginning of the intervention (as baseline assessment), immediately after the first session of virtual training (as short-term assessment), and at the end of the entire intervention (as long-term assessment). We also recorded mood changes (with the Two-Dimensional Mood test) before and after each virtual session.

We hypothesized the following: (1) to replicate previous findings on body ownership/agency and physiological effect in the elderly population—meaning the 1PP group experiences ownership and agency over the avatar, which leads to increased heart rate coherently with the virtual movements, while the 3PP group does not; (2) to replicate previous findings on acute cognitive benefits—meaning the cognitive performance is improved immediately after the first session of virtual training in 1PP combined with an increased activity over the lDLPFC, confirming the acute cognitive benefits of this training also on the elderly; and (3) to find an increased cognitive improvement after the 6-week training in the 1PP group of elderly and not in the 3PP group.

## Materials and Methods

**FIGURE 1:**
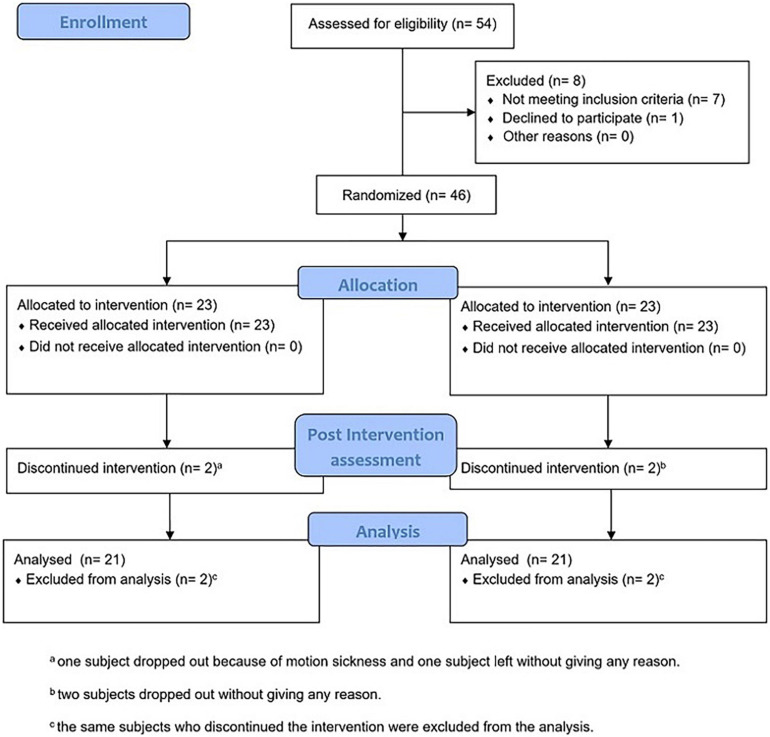
CONSORT flow diagram of the present registered clinical trial (UMIN000039843).

The protocol of this parallel-group RCT, developed according to CONSORT guidelines (Figure 1) and carried out in accordance with the Declaration of Helsinki, was registered to the University Hospital Medical Information Network (UMIN) Clinical Trial Registry (UMIN000039843) on March 18, 2020 and approved by the Ethics Committee of the Tohoku University Graduate School of Medicine (application no. 2019-1-956, final approval April 9, 2020). As soon as the participants visited the laboratory for the first session, in addition to a verbal explanation of the entire experimental procedure, each of them signed the information sheet and the informed consent form before the initiation of the study, agreeing to the conditions of their participation. The raw data that support the findings of this study are available upon request to the corresponding author.

### Participants

We recruited the participants through an advertisement published in a local newspaper (Sendai, Japan), so they were Japanese nationals and native Japanese speakers. A total of 54 people were first screened *via* phone call: we excluded under 60 years old, those who had a history of neurological, psychiatric, or motor disorders, and color blindness, and we asked to refrain from participating those who easily experience motion sickness or dizziness. Eight of them did not meet the inclusion criteria or did not accept to the experiment’s conditions; therefore, they were excluded. We initially recruited and allocated 46 subjects. While the RCT was ongoing, three participants dropped out for no explicit reason (one of them was in the experimental group) and one dropped out because of motion sickness (this subject was part of the experimental group).

Forty subjects entered and completed the entire RCT and were included in the analysis (28 females; age: average = 71.71 years, SD = 5.71 years; education: average = 13.85 years, SD = 2.24 years). In the Edinburgh Handedness Inventory, they all resulted right handed (average = 96.21, SD = 9.09). In the Physical Activity Questionnaire (IPAQ—Short Form), they all resulted with a score from “moderate” to “high” physical activity level, indicating their general health and engagement in physical activity (the subjects who scored “moderate” were 13 in the 1PP group and 14 in the 3PP group).

After enrollment, the participants were then randomly allocated to one of two study arms: the first-person perspective group (hereinafter, 1PP group), the experimental one, and the third-person perspective group (hereinafter, 3PP group), the control one. Group assignment occurred using a simple randomization 1 (experimental):1 (control) ratio, with the allocation of participants to each arm based on order of entry into the study (Summers et al., 2018).

The demographic composition of the groups is as follow: in the 1PP group, there are 21 subjects (11 females; age: average = 70.57 years, SD = 6.51 years; education: average = 14.25, SD = 2.46); in the 3PP group, there are also 21 subjects (17 females; age: average = 72.86 years, SD = 4.65 years; education: average = 13.45 years, SD = 1.95 years). The *t* test comparing the age and education of the two groups resulted not significant (age: *p* = 0.19; education: *p* = 0.26). A chi-square test of independence showed that there was a significant difference between gender and group [*χ*^2^(1,42) = 3.85, *p* = 0.04; Yates-corrected *χ*^2^ = 2.68, *p* = 0.10]. Despite this, we proceeded with the 1:1 ratio group assignment since we did not have any assumptions related to the main outcome of the study regarding gender.

The sample size was estimated using G^∗^Power 3.1 with *a priori* power analysis for an *F* test with between–within interactions: considering the Stroop task’s response time as the main outcome, we set a small to moderate effect size [*f*(*V*) = 0.4] (Byun et al., 2014; Monteiro-Junior et al., 2017a; Burin et al., 2020), so we calculated a total sample size of 52 subjects (with the α error probability set at 0.05 and power set at 0.8). We were able to recruit 46 subjects.

### Procedure

**FIGURE 2:**
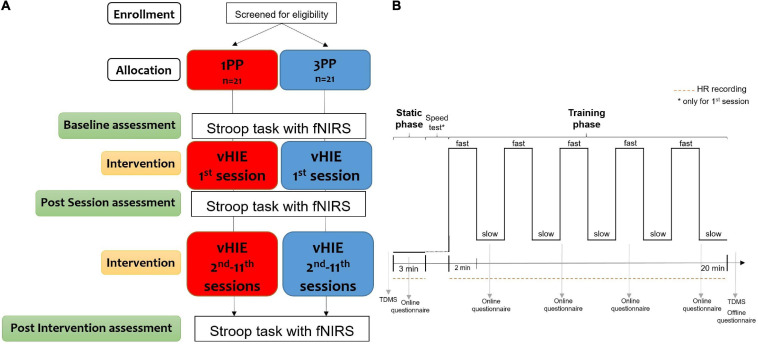
Schematic procedure of the randomized controlled trial (RCT) **(A)** with the timeline and measurements for each session of the virtual high-intensity intermittent exercise (vHIE) intervention **(B)**. After recruitment, the subjects were verbally screened, according to the inclusion criteria for this study, and then allocated to one of two groups [first-person (1PP) or third-person (3PP) perspectives]. During the first session only (which lasted approximately 1 h), all participants read and signed the information sheet and consent form, and they received an additional verbal explanation of the entire RCT procedure, giving them the possibility to ask questions. They also filled out the Edinburgh Handedness Inventory and the International Physical Activity Questionnaire (IPAQ—Short form) for handedness and level of physical activity, respectively. As baseline assessment, they all underwent the color–word matching Stroop task during the recording of cortical hemodynamic changes with a functional near-infrared spectroscopy (fNIRS) device over the left dorsolateral prefrontal cortex (lDLPFC). Then, the intervention started **(B)**: all participants completed the Two-Dimensional Mood Scale (TDMS) including the two subscales for pleasure and arousal. After that, they performed for the first time the vHIE (with the online recording of the heart rate and subjective questionnaire about sense of ownership and agency) in the 1PP or 3PP, according to the group allocation. Immediately after that, they filled out a questionnaire about their experience and they repeated the TDMS. As post-session assessment, they all underwent again the Stroop task with fNIRS recording **(A)**. The procedures of the sessions from the second to the 11th were the same (each lasted about 30 min): according to the group allocation, participants experienced the vHIE in the 1PP or the 3PP, with the online recording of the heart rate and questionnaires during and right after the vHIE part. All subjects repeated the TDMS before and after the vHIE for each session **(B)**. As post-intervention assessment, during the 12th session (which lasted about 15 min), all subjects repeated the Stroop task with the fNIRS recording **(A)**.

The RCT protocol was composed of 12 separate sessions (Figure 2). We invited participants to visit the laboratory twice a week (for example, every Monday and Thursday or every Tuesday and Friday, compatible with their availability) for consequent weeks, without interruption, to maintain as constant as possible the duration and timing of the intervention (Mirelman et al., 2016; Monteiro-Junior et al., 2017b): the volunteers carried on the actual sessions on average 3.54 days (SD = 0.25 days) between each other; this resulted in an average of 38.31 days (SD = 2.34 days) between the first and the last session.

#### Virtual High-Intensity Intermittent Exercise

The IVR setup used in this study was the same as that already tested in Burin et al. (2020), with the exceptions that, here, the RCT is a parallel-group design and the same intervention is repeated for 11 sessions for 20 min each (Mirelman et al., 2016; Monteiro-Junior et al., 2017b).

**FIGURE 3:**
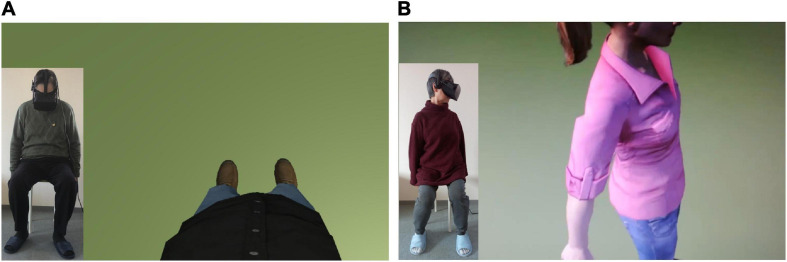
Virtual bodies in the virtual scenario. **(A)** The male virtual body displayed in a first-person perspective (1PP) from the same perspective of the participant (in the *bottom left corner*, the male participant is sitting and looking down toward himself, where his virtual body is). **(B)** The female virtual body displayed in a third-person perspective (3PP) from the perspective of the participant (in *the corner*, the female participant is sitting and looking to the left side at the virtual body).

During the vHIE part of each session, the participants were instructed to sit and not to move their bodies, with their feet resting on the ground and their arms relaxed along the body side. However, they were allowed to move their neck and rotate their head in order to always look at the virtual body (Figure 3). Through the Oculus Rift visor^1^, they saw a virtual environment, modeled in Unity3D, composed of a simple open space with a green floor (simulating a meadow) and a natural-like bright sky. The gender-matched life-sized humanoid standing bodies were downloaded from the Microsoft Rocketbox Avatar public library (Gonzalez-Franco et al., 2020).

The intervention performed by the two groups was the same, except for the crucial difference made by the visual perspective: in the 1PP group, the virtual body was displayed in a first-person perspective, coherently with the real one, spatially overlapping it (Figure 3A), known to be the crucial condition to induce a sense of ownership and to create the virtual full-body illusion (Kokkinara et al., 2016; Burin et al., 2019a, 2020; Neyret et al., 2020). In the 3PP group, the virtual body was located about 1.5 m to the left of the actual participant’s position, resembling another person (Figure 3B) and not inducing the same illusion mentioned in the 1PP group (Pavone et al., 2016; Gonzalez-Liencres et al., 2020).

The vHIE part was repeated for all sessions, except for the last one (where subjects did only the post-intervention assessment), with the same characteristics (Figure 2B): for the first 3 min, the virtual body is displayed standing, either in 1PP or 3PP, but it does not move (hereinafter, static phase) in order to familiarize with the environment, to eventually control for dizziness or sickness due to the virtual display and to induce the illusion of a sense of ownership thanks to the perspective, considering that it may take a few seconds/minutes for the subjective perception, as it happens with other multisensory illusions (Burin et al., 2018). For the following 20 min (hereinafter, training phase), the virtual body in both conditions (1PP and 3PP) alternates 2 min of fast walking (also called fast phase) and 2 min of slow walking (also called slow phase) (Kokkinara et al., 2016; Kujach et al., 2017), while the participant is sitting still. Right after the static phase of the first session only, the participants were asked to choose a speed for the fast walking animation that would be appropriate for them among four options (1: 3.30 m/s, 2: 4 m/s, 3: 4.30 m/s, and 4: 5 m/s), while the speed for the slow walking animation was the same for all subjects (0.5 m/s). Then, the chosen speed for the fast walking parts was kept constant for the following sessions for each subject. As previously done (Burin et al., 2020), this procedure ensured (especially in the experimental group) that the fast walking animation was subjectively reported as considerably fast (in order to show a detectable physiological activation), but not too much to be impossible to perform (in order to not break the ownership and agency illusion).

We decided that the duration of each training phase was 20 min based on a previous report (Monteiro-Junior et al., 2017b), but also for safety reasons: because it is not a medical device but is considered an entertainment system, there are no international safety guidelines on the use of IVR devices. Based on previous studies and the experience of the researchers, we decided to keep the duration of the vHIE part no longer than 30 min (Hamilton et al., 2021).

For the entire duration of the vHIE part of the session, we repeatedly asked the participants to immediately report any feeling of discomfort, nausea, sickness, etc. As previously described, one subject (belonging to the 1PP group) reported nausea during his/her fourth session; consequently, the intervention was interrupted (his/her data were discarded and not included in the analysis reported here).

##### Heart rate

For the entire duration of the vHIE part of each session (composed of the static and training phases) and for both groups, we recorded the heart rate (HR): the increased physiological activation, even in static conditions, might be a measurable reflection of the anticipation or preparation of the body to move, as it happens with motor imagery studies (Wegner and Wheatley, 1999), or a direct effect of the sense of agency over a moving body performing a physical task that requires physiological activation (Kokkinara et al., 2016; Burin et al., 2020). In addition, the recording of the HR in this study was necessary to validate the actual presence of the virtual illusion (especially in the 1PP group) with an objective measurement, in addition to the subjective component (see Section “Online Questionnaire on Sense of Body Ownership and Agency”), and to check the effectiveness of the training itself. As previously done (Burin et al., 2020), we used a Polar H10 (Polar Electro, Kempele, Finland; polar.com), a very common heart rate monitor used by athletes, connected *via* Bluetooth to a smartphone where an *ad hoc* application collects the recorded data (flow.polar.com). The HR monitor was pinned to an elastic strip worn around the chest, before the beginning of the vHIE part every session, positioning it as close as possible to the heart.

##### Online questionnaire on sense of body ownership and agency

The questionnaires administered in this study are the same as that previously used in Burin et al. (2020). During the static and training phases of the vHIE for each session, we administered an online questionnaire in order to check the effectiveness of the virtual illusion from a subjective perspective, referring specifically to the illusory sense of body ownership and agency over the virtual body. The online questionnaire was verbally administered by the researchers and the subjects had to report their level of agreement with the questionnaire’s statements on a 1–7 Likert scale (1 = meaning “totally disagree” and 7 = meaning “totally agree”). The questionnaire included four statements (from s1 to s4 in Table 1), two of them about the sense of body ownership and two about the sense of agency (for each, one is a “real statement” that checks for the actual presence of the illusion, while the other is a “control statement”) (Table 1). The same statements were repeated in a random order at five time points: at 1 min and 30 s after the beginning of the static phase and at 3, 8, 13, and 18 min after the beginning of the training phase, for every single session. This repetition throughout the session was necessary to eventually check for differences in the fluctuation of the illusion (especially in the 1PP group) and potential changes between the fast (minutes 8 and 13 of the training phase) and slow (minutes 3 and 18 of the training phase) phases.

**TABLE 1:** Online subjective questionnaire verbally administered during the virtual high-intensity intermittent exercise (vHIE) for each session and group.

Online questionnaire				1PP group: average ± SE	3PP group: average ± SE	*p* value
Static phase	s1	Sense of body ownership	*I feel as if I am looking at my own body.*	4.69 ± 0.39	2.04 ± 0.32	< 0.01*
	s2	Sense of body ownership control	*I feel as if the virtual body belongs to another person.*	3.40 ± 0.40	6.08 ± 0.29	< 0.01*
	s3	Sense of agency	*The virtual body moves just as I want, as if I am controlling it.*	3.63 ± 0.36	1.89 ± 0.31	< 0.01*
	s4	Sense of agency control	*I feel as if the virtual body is controlling my will.*	2.30 ± 0.26	1.48 ± 0.17	0.57
Training phase	s1	Sense of body ownership	*I feel as if I am looking at my own body.*	4.74 ± 0.41	2.01 ± 0.34	< 0.01*
	s2	Sense of body ownership control	*I feel as if the virtual body belongs to another person.*	3.37 ± 0.42	6.13 ± 0.31	< 0.01*
	s3	Sense of agency	*The virtual body moves just as I want, as if I am controlling it.*	3.69 ± 0.37	1.88 ± 0.31	< 0.01*
	s4	Sense of agency control	*I feel as if the virtual body is controlling my will.*	2.29 ± 0.29	1.49 ± 0.17	0.81

The columns, from left to right, indicate respectively the phase during which the questionnaire was administered, the statement number (for example, s1), the underlying domains (not disclosed to subjects) and the actual statements (in italic). The results for the first-person perspective (1PP) and third-person perspective (3PP) groups are expressed as average ± standard error. The data in the table are expressed on the 1–7 Likert scale (not ipsatized data) and each statement is averaged across all the sessions of the intervention. *Significant *p* values comparing groups.

##### Offline questionnaire on sense of ownership and agency

Right after the vHIE part of each session, the participants were asked to complete another questionnaire with more detailed statements about subjective feelings of movements, motor control, and physical effort. This questionnaire was self-administered. As mentioned before, the statements here are the same as those of Burin et al. (2020), adapted from Kokkinara et al. (2016) and Burin et al. (2019a) (see Table 2).

**TABLE 2:** Offline subjective questionnaire self-administered immediately after the virtual high-intensity intermittent exercise (vHIE) part for each session in both groups.

Offline questionnaire			1PP group: average ± SE	3PP group: average ± SE	*p* value
s5	Located	*I felt as if my body was located where I saw the virtual body to be.*	4.37 ± 0.33	2.49 ± 0.40	0.02*
s6	Sense of ownership	*I felt that the virtual body was my own body.*	4.15 ± 0.39	2.27 ± 0.37	0.01*
s7	Standing	*I felt that I was standing upright.*	3.98 ± 0.39	2.37 ± 0.31	0.16
s8	My movements	*I felt that the leg movements of the virtual body were my movements.*	4.21 ± 0.39	2.41 ± 0.39	0.02*
s9	Sense of agency	*I felt that the leg movements of the virtual body were caused by my movements.*	3.59 ± 0.30	2.49 ± 0.29	0.23
s10	Sense of ownership control	*I felt that the virtual body belonged to someone else.*	3.69 ± 0.32	4.82 ± 0.35	< 0.01*
s11	Effort	*I felt I had to give extra physical effort when the virtual body was walking faster.*	3.75 ± 0.34	2.12 ± 0.25	0.04*
s12	Vection	*I felt that I was moving through space rather than the world moving past me.*	4.72 ± 0.36	2.58 ± 0.38	0.01*
s13	Walking	*I felt that I was walking.*	4.13 ± 0.33	2.45 ± 0.37	0.04*
s14	Dragged	*I felt that I was being dragged.*	1.79 ± 0.16	1.56 ± 0.17	< 0.01*
s15	Sliding	*I felt that I was sliding.*	2.04 ± 0.19	1.69 ± 0.18	< 0.01*

The columns, from left to right, indicate respectively the statement number (for example, s5, continuing from previous ones), the underlying domains (not disclosed to subjects) and the actual statements (in italic). The results for the first-person perspective (1PP) and third-person perspective (3PP) groups are expressed as average ± standard error. The data in the table are expressed on the 1–7 Likert scale (not ipsatized data) and each statement is averaged across all the sessions of the intervention. *Significant *p* values comparing groups.

We decided to repeat the questionnaires for each session to control for potential effects of time (meaning, the illusion’s strength might be different between sessions).

##### Two-dimensional mood scale

Before and after the vHIE part for each session, the participants completed the Two-Dimensional Mood Scale (TDMS) to record mood state changes that might affect physiological responses (e.g., the heart rate). TDMS includes two subscales: pleasure and arousal (Sakairi et al., 2013). The participants rated their current psychological state using a six-point Likert scale from 0 = “Not at all” to 5 = “Extremely.”

#### Baseline, Post-session, and Post-intervention Assessments

The baseline assessment coincided with the beginning of the first session, while the post-session assessment was performed after the first repetition of the vHIE part. Lastly, the post-intervention assessment coincides with the very end of the entire vHIE intervention, so it was performed during the last (12th) session, an average of 3.35 days (SD = 1.99 days) after the last repetition of the vHIE part (Figure 2A).

The baseline assessment ensured evaluating the starting level abilities for each person and checking the actual presence of the typical Stroop effect in the sample; then, the comparison with post-session and post-intervention evaluates the short-term and the long-term effects of the vHIE intervention. The assessment was the same for all of the participants: we used the Stroop task to test executive functions, with the online recording of cortical hemodynamic changes over lDLPFC.

##### Stroop task

Like we did in Burin et al. (2020), the Stroop task used was developed in E-prime 2.0 and was administered and recorded automatically from a laptop. It includes 30 trials presented in random order. For each single trial, two words are displayed on the PC monitor, one above the other: for the 10 neutral trials, the upper row consists of XXXX printed in red, white, blue, brown, or yellow ink, and the lower row shows the words “RED,” “WHITE,” “BLUE,” “BROWN,” or “YELLOW” printed in black. For the 10 congruent trials, the upper row contains the same words printed coherently in the same color (e.g., RED written in red), and the lower row shows the same words printed in black. For the 10 incongruent trials (the ones that produce cognitive interference between the color word and the color name, i.e., Stroop interference), the word in the upper row is printed in an incongruent color (e.g., RED written in yellow). All words were written in Japanese hiragana (except for XXXX). The lower row is presented 100 ms later than the upper row to achieve sequential visual attention. Between each trial, an inter-stimulus fixation cross is shown for a random interval between 9 and 13 s to avoid prediction (Hyodo et al., 2012; Byun et al., 2014; Kujach et al., 2017). The words remain on the screen for 3 s, independently of the subject’s answer. Subjects were instructed to decide whether the color of the upper word (or XXXX) corresponded to the color name of the lower word by pressing button 1 on the keypad to give a “yes” or button 2 a “no” response with their right forefingers. Fifty percent of the presented stimuli were correct (the correct answer is “yes”).

##### Functional near-infrared spectroscopy

While performing the Stroop task during the baseline, post-session, and post-training assessments, the participants wore a wearable fNIRS optical topography system (WOT-HS, Hitachi Corporation and NeU Corporation, Japan) managed by its software (Hitachi Solutions, Inc.). This system is the same as that used in the previous study (Burin et al., 2020): the 35 capsules of this device compress near-infrared emitting or high-sensitivity receiving sensors, organized in three lines (the top and the bottom lines alternate an emitting and a receiving sensor, while the central line comprises receivers only), creating a system of 34 channels over the lateral and anterior prefrontal cortex. The device was positioned on the forehead by centering the specific mark on the bottom line of probes at the frontopolar zone (FPZ; 10% of the distance between the nasion and inion), according to the international 10–20 system (Klem et al., 1999).

Various previous studies have stressed the importance of the prefrontal cortex (PFC) and specifically the dorsolateral prefrontal cortex (DLPFC) in the context of the executive performance (MacDonald et al., 2000; Hoshi et al., 2001). More specifically, because of the significance of the lDLPFC in relation to the executive performance examined, thanks to the Stroop task (Yanagisawa et al., 2010; Hyodo et al., 2012, 2016), and the similarity between the previously used tasks and the present one (Kujach et al., 2017; Burin et al., 2020), we focused the analysis on lDLPFC. To monitor the cortical hemodynamic changes in the lDLPFC, we recorded the concentrations of oxygenated hemoglobin (O_2_Hb) and deoxygenated hemoglobin (HHb), expressed in units of millimolar.millimeter (Watanabe et al., 1995), by applying two short-distance wavelengths of near-infrared light (850 and 730 nm).

### Statistical Analysis

For all analyses, distribution was assessed using a Shapiro–Wilk test for normality and, accordingly, parametric or non-parametric analyses were conducted. The significance level was set at *p* < 0.05. *Post hoc* analysis was conducted with Duncan’s test. All displayed values are average ± standard error (SE). When necessary, we performed a retrospective power analysis (between-group effect sizes using G-Power) with specified effect sizes: Cohen’s *d* was used for parametric comparisons, while for non-parametric eta squared (η^2^) was used (with α error probability set at 0.05).

#### Heart Rate Data

The software recorded the HR data as the instantaneous heart rate changes (expressed in beats per minute, as in Kokkinara et al., 2016; Burin et al., 2020) at 1 Hz frequency (Malik, 1996) during the static (3 min) and the training (20 min) phases of each session. At first, we excluded from the individual raw data outliers or artifacts, defined as data with values ± 20% or greater with respect to the adjacent one (Ribeiro et al., 2018). For each session, the  HR recordings (23 min in total) were  divided into the corresponding phases  of the vHIE part, i.e., static (one segment, corresponding to  the first 3 min), fast walking  (five segments of 2 min each), and slow  walking (other five segments of  2 min each, temporally alternated between fast walking and slow walking) phases. For each segment accordingly obtained, we discarded the first 30 s of recordings: this procedure ensured considering in the analysis the actual HR variability directly imputable to the IVR stimulation, considering that HR is a slow physiological measurement that requires time to adapt to external events (Wang et al., 2018). Finally, for each subject, we averaged the obtained segments corresponding to the same phase (static, fast walking, and slow walking). Lastly, we subtracted the obtained data for static (HRst) from the data of fast walking (HRf) and slow walking (HRs), resulting in dHRf (= HRf - HRst) and dHRs (= HRs - HRst) for each group.

Since data were not normally distributed, we ran a Mann–Whitney *U* test comparing the two groups.

#### Online Questionnaire Data

The four online questionnaire statements (from s1 to s4; see Table 1) have been repeated in a random order during each session at five time points (during the static phase at 1 min 30 s and during training at 3, 8, 13, and 18 min). The obtained raw data from each statement underwent an intra-individual ipsatization procedure (Cattell, 1944): this is a quite common procedure (also called “standardization per person”) that applies to subjective measurements (such as questionnaires), and it allows neutralizing potential response biases in a response set. The ipsatization was done as follows: every raw value was first subtracted by the mean rating of the subject responses in all questions and conditions and then divided by the standard deviation of the responses in all questions and conditions (Romano et al., 2014; Burin et al., 2015), obtaining *z*-scores ± SE. Although the analysis was performed with *z*-scores, in Table 1, we reported the non-ipsatized data (average ± SE) in order to have a more precise reference of the answers on the 1–7 Likert scale.

In test *W*, the data resulted as non-normally distributed, so we ran a Mann–Whitney *U* test comparing each statement separately (separating also the static and training phases) between groups.

#### Offline Questionnaire Data

The 11 statements of the offline questionnaire (from s5 to s15; see Table 2) were repeated right after the end of the vHIE part of each session. As for the online ones, the answers to the offline questionnaire were ipsatized (Pia et al., 2015), but in Table 2, we reported non-ipsatized data. The data resulted as non-normally distributed, so we ran a Mann–Whitney *U* test comparing each statement (from s5 to s15) by groups (1PP and 3PP).

#### TDMS Data

According to the TDMS guidelines, we calculated pleasure and arousal levels separately (Sakairi et al., 2013). We ran a 11 × 2 × 2 ANOVA with factors session (corresponding to the 11 sessions), time (pre and post each session), and group (1PP and 3PP) for each pleasure and arousal data.

#### Stroop Task’s RT and ER Data

We recorded as outcomes the response time (RT, in milliseconds), as the difference between the display of the upper row stimulus and the subject having given an answer, and the error rate (ER, in percentage of error; missed trials or answered over the time limit are considered errors).

Concerning the RT measurements of the Stroop task (main outcome of this study), we first compared the ones recorded during the baseline assessments. In test *W*, all RT data resulted as normally distributed, so we ran a 2 × 2 repeated measures ANOVA with factors condition (neutral and congruent) and group (1PP and 3PP). The Stroop task’s crucial outcome is the so-called Stroop interference, which is assumed to actually characterize the cognitive process underlying the task itself, defined as the average of incongruent trials - average of neutral trials (Zysset et al., 2001). Hence, the 2 × 3 repeated measures ANOVA with between factor group (1PP and 3PP) and within factor time of assessment (baseline, post-session, and post-intervention).

Concerning the ER measurements (expressed in percent of error) of the Stroop task, we first controlled again whether there was the typical Stroop interference effect in the sample. In this case, we ran a Wilcoxon matched pair test to compare the ER results during the baseline assessment for all subjects (independently of group assignment). Mainly, we compared with Mann–Whitney *U* test the ER between groups (1PP and 3PP) and the time of assessment (baseline, post-session, and post-intervention).

#### fNIRS Data

**FIGURE 4:**
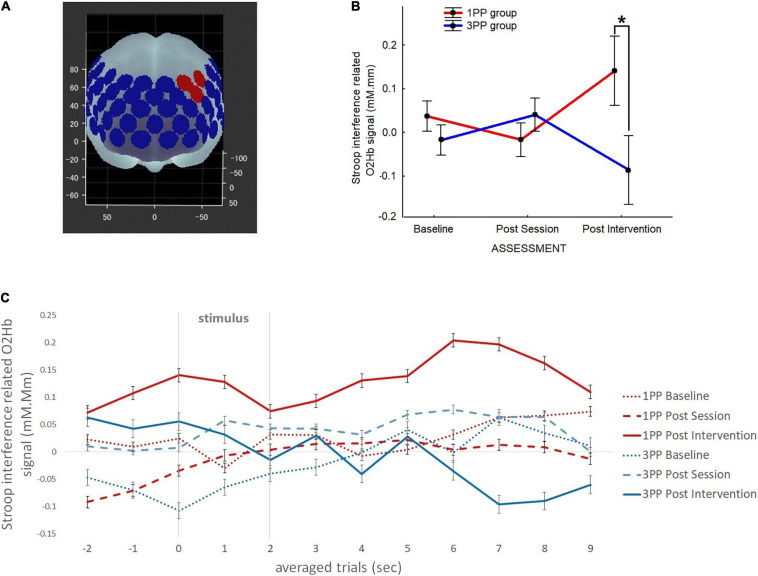
Line plots of the functional near-infrared spectroscopy (fNIRS) Stroop interference-related results over the left dorsolateral prefrontal cortex (lDLPFC) for the oxygenated hemoglobin (O_2_Hb) signal. **(A)** Representation of the fNIRS device’s channels over the prefrontal cortex: *red spots* (representing channels 23, 25, and 26) are the ones considered for the analysis of the lDLPFC (channel 23: *x* = -30, *y* = 33, *z* = 51; channel 25: *x* = -47, *y* = 16, *z* = 50; channel 26: *x* = -43, *y* = 34, *z* = 39). **(B)** Results of the Stroop interference-related activation of the O_2_HB signal (in millimolar.millimeter) across the three assessment time points: baseline, post-session, and post-intervention. *Asterisk* highlights the significant differences (*p* < 0.05). *Red line* refers to the results of the first-person perspective (1PP) group, while *blue line* refers to the third-person perspective (3PP) group. *Vertical black bars* denote plus/minus standard errors. **(C)** fNIRS data (O_2_HB signal only) associated with Stroop interference activation showing the timeline of the averaged trials per group and assessment. *Red lines* refer to the results in the 1PP group, while *blue lines* refer to those of the 3PP group. *Dotted lines* show results recorded during the baseline assessment, *dashed lines* refer to post-session, and *solid lines* are for post-intervention assessment. The *X*-axis displays the time in seconds for every trial of the Stroop task: 2 s before the stimulus display, the stimulus is displayed (from 0 to 2 s) and the next 7 s (where there is an increased peak around 6–8 s in the 1PP post-intervention line). *Vertical black bars* denote plus/minus standard errors.

The optical fNIRS data of the O_2_Hb and HHb signals (sampling rate at 10 Hz) were analyzed according to the modified Beer–Lambert law (Delpy et al., 1988). After processing each channel singularly (see Burin et al., 2019c for details), we focused on channels 23, 25, and 26, which are associated with the target area, lDLPFC (Figure 4A). Because of the time difference between the fNIRS signals of participants’ responses, we selected for each trial the averaged changes in the concentrations of O_2_Hb and HHb 2 s before the onset task as a “rest,” during the “task” (lasting for 3 s), and 10 s after the onset task as “vascular response” (Schroeter et al., 2002). In an event-related design, we matched each trial’s signal with the corresponding Stroop task conditions and averaged them. As for the Stroop task results, we considered here the Stroop interference (incongruent–neutral condition).

O_2_Hb and HHb (expressed in millimolar.millimeter) were analyzed separately by means of a 2 × 3 ANOVA, with group (1PP and 3PP) as the categorical factor and time of assessment (baseline, post-session, and post-training) as the within-subjects factor.

#### Correlations Analysis

Lastly, we checked for correlations among the above-mentioned variables. Because of the elevated number of independent correlations, we also applied a false discovery rate (FDR) procedure with α = 0.05 (Benjamini and Hochberg, 1995), so we displayed the FDR-adjusted *p* value for each correlation.

At first, we tested for correlations during the vHIE part, i.e., HR and data of the online questionnaire. Considering each statement separately (from s1 to s4), we compared the HR results from the training (fast walking and slow walking phases) with Spearman’s correlation; then, we ran the same analysis for the correlations between HR and the offline questionnaire. Secondly, we correlated the data collected during the assessments, i.e., Stroop task’s RT and ER with O_2_Hb signal, with Pearson’s correlation. Lastly, we correlated the measurements during the vHIE (HR and questionnaires) with the measurements during the assessments (RT and ER of Stroop interference and the O_2_Hb signal) with Spearman’s correlation.

## Results

To test hypothesis 1 about the subjective and physiological effects of the illusory virtual body on the real one (see Section “Introduction”), we analyzed first the data from the online and offline questionnaires and the heart rate data. Then, to test hypotheses 2 and 3, we proceeded with the main analysis of the Stroop test, fNIRS, and TDMS measurements across the three assessment time points. Lastly, we ran correlations among them.

### Heart Rate

Considering that we did not find differences in time, i.e., the main effect of session, or in the interaction with group, we averaged the results across sessions for each subject.

**FIGURE 5:**
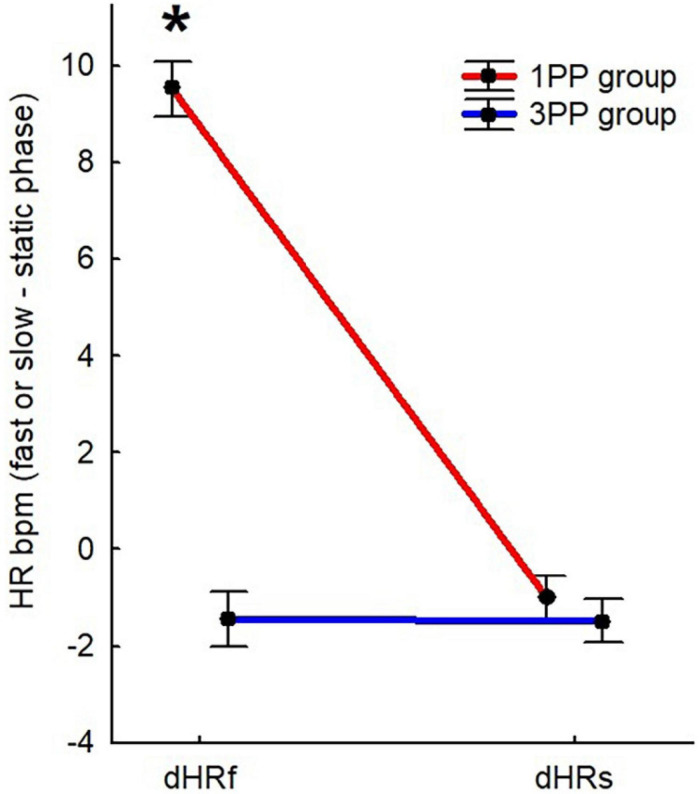
Line plots of the heart rate (HR) results (expressed in beats per minute). *Red line* represents the results of the first-person perspective (1PP) group and *blue line* is for the results of the third-person perspective (3PP) group. *Left part of the graph* shows the results of HR during the fast phase (dHRf) of the virtual high-intensity intermittent exercise (vHIE) across sessions and the *right one* concerns the slow phase (dHRs). For both, the results shown here are obtained by subtracting the corresponding static phase. Significant differences (*p* < 0.05) are highlighted with *asterisk*. *Vertical black* bars denote plus/minus standard errors.

In the Mann–Whitney *U* test comparing the two groups dHRf was significantly (*p* < 0.01, 2^∗^1-sided exact *p* < 0.01, adjusted *z* = 5.27) higher in the 1PP (9.53 ± 0.57) with respect to the 3PP (−1.44 ± 0.57) group. The dHRs did not result significantly different in the group comparison (1PP: −0.99 ± 0.45; 3PP: −1.47 ± 0.449) (Figure 5). The retrospective power analysis resulted in power = 0.95, η^2^ = 0.80.

As previously mentioned, the participants were allowed to choose a speed for the fast walking phase animation appropriate for them: 41 subjects chose speed 1 (3.30 m/s) and only one subject (in the 1PP group) chose speed 2 (4 m/s).

### Online Questionnaire on Sense of Body Ownership and Agency

Firstly, we checked eventual differences between sessions (meaning that ratings to the same statement do not change across the time of the intervention) and also in the repetitions of the statements in the training phase (meaning that ratings to the same statement do not change across the time of the same session). We found no relevant differences between sessions or within the sessions, so we averaged for each subject each statement separately across sessions for the static phase and also across repetitions in the same session for the training phase (see Supplementary Material).

**FIGURE 6:**
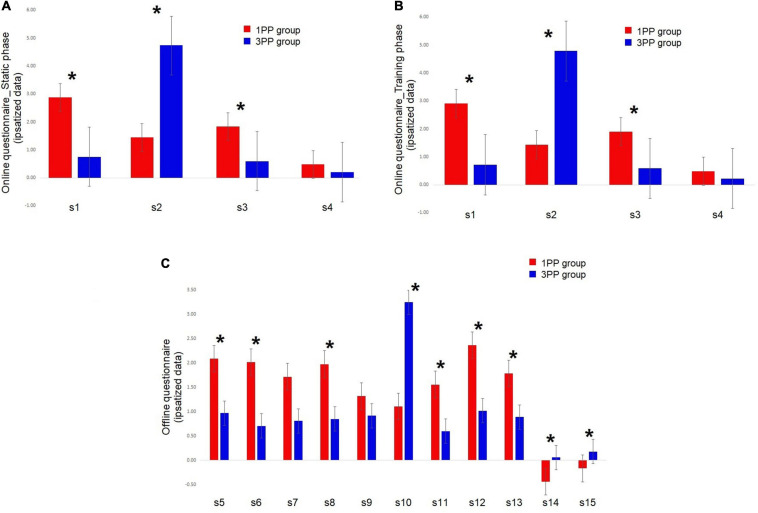
Histograms of the online and offline questionnaires about sense of body ownership and agency results (ipsatized data used in the analysis). **(A)** Results of the online questionnaire (from s1 to s4) during the static phase. **(B)** Results of the online questionnaire (from s1 to s4) during the training phase (averaged between repetitions). **(C)** Results of the offline questionnaire (from s5 to s15). All the displayed data represent the average scores between sessions. *Red bars* represent the results of the first-person perspective (1PP) group and *blue bars* the results of the third-person perspective (3PP) group. Significant differences (*p* < 0.05) are highlighted with *asterisk*. *Vertical black bars* denote plus/minus standard error.

In the Mann–Whitney *U* test comparing groups, in the static phase, s1 (about sense of body ownership) was significantly different (*p* < 0.01, 2^∗^1-sided exact *p* < 0.01, adjusted *z* = 3.81) between the 1PP group (2.87 ± 0.33) and the 3PP group (0.75 ± 0.33), with 1PP higher than the control group. The same pattern was found for s3, the statement about sense of agency (*p* < 0.01, 2^∗^1-sided exact *p* < 0.01, adjusted *z* = 2.88), with ratings in the 1PP group (1.84 ± 0.31) higher than those of 3PP (0.59 ± 0.31). In contrast, s2, the control statement on body ownership, showed the opposite pattern: in 1PP (1.44 ± 0.44), s2 was significantly lower (*p* < 0.01, 2^∗^1-sided exact *p* < 0.01, adjusted *z* = -4.29) than that in the 3PP group (4.74 ± 0.44). Lastly, s4, the control statement about agency, did not differ between groups (Figure 6A).

In the training phase, it seems that the pattern of ratings in the static phase is maintained. s1, about sense of body ownership, was significantly (*p* < 0.01, 2^∗^1-sided exact *p* < 0.01, adjusted *z* = 3.68) higher in the 1PP group (2.91 ± 0.36) than in the 3PP group (0.72 ± 0.36)., The same goes for s3, the statement about sense of agency (*p* < 0.01, 2^∗^1-sided exact *p* < 0.01, adjusted *z* = 2.95), which was, again, higher in the 1PP group (1.90 ± 0.32) than in the control group (0.59 ± 0.32). The control statement s2, the statement on body ownership, was significantly (*p* < 0.01, 2^∗^1-sided exact *p* < 0.01, adjusted *z* = -4.21) lower in the 1PP group (1.43 ± 0.45) than in 3PP (4.79 ± 0.45) (Figure 6B). Again, s4, the control statement about agency, did not differ between groups. The retrospective power analysis resulted in power = 0.85, η^2^ = 0.73.

Despite the above-mentioned significances, if we consider the non-ipsatized data (see Table 1), in the 1PP group, s1 (statement about body ownership) during the static phase was rated 4.69/7 and during the training phase was rated 4.74/7, meaning close to “I slightly agree.” s3 (about sense of agency) in the static phase was rated 3.63/7 and in the training phase was 3.69/7, meaning close to “I don’t know” (corresponding to 4/7). If we consider the groups separately and compare the statements (with Wilcoxon test), in the 1PP group, s1 (about sense of body ownership) during the static and also the training phase was not significantly different from its control (s2), while s3 (about sense of agency) was significantly higher (static: *p* < 0.01, *z* = 2.69; training: *p* < 0.01, *z* = 2.66) than s4 (its control statement) in static (2.30/7) and training (2.29/7).

### Offline Questionnaire on Sense of Body Ownership and Agency

Firstly, we needed to check whether there was an effect of the session (meaning that ratings to the same statement do not change across the time of the intervention): we found no main effect of sessions or interaction with statement or group, so we proceeded by averaging the answers to the same statement across sessions.

In the Mann–Whitney *U* test comparing between groups, s5, about sense of being located where the virtual body was, was significantly different (*p* = 0.01, 2^∗^1-sided exact *p* = 0.02, adjusted *z* = 2.35) and higher in the 1PP group (2.08 ± 0.32) with respect to the 3PP group (0.92 ± 0.27). The same pattern was found for s6, about sense of body ownership (*p* = 0.01, 2^∗^1-sided exact *p* = 0.01, adjusted *z* = 2.52; 1PP: 2.01 ± 0.36; 3PP: 0.70 ± 0.24); s8, about the ownership of the virtual movements (*p* = 0.02, 2^∗^1-sided exact *p* = 0.02, adjusted *z* = 2.20; 1PP: 1.97 ± 0.37; 3PP: 0.85 ± 0.27); s11, about sense of effort (*p* = 0.03, 2^∗^1-sided exact *p* = 0.04, adjusted *z* = 2.07; 1PP: 1.55 ± 0.31; 3PP: 0.60 ± 0.18); s12, concerning the feeling of the movement of the body in the space, or vection (*p* = 0.01, 2^∗^1-sided exact *p* = 0.01, adjusted *z* = 2.47; 1PP: 2.36 ± 0.344; 3PP: 1.01 ± 0.28); and s13, about the feeling of walking (*p* = 0.04, 2^∗^1-sided exact *p* = 0.04, adjusted *z* = 2.05; 1PP: 1.78 ± 0.30; 3PP: 0.89 ± 0.25). The opposite pattern was shown in s10, the control statement about sense of body ownership, where ratings in the 1PP group (1.10 ± 0.37) were significantly lower (*p* < 0.01, 2^∗^1-sided exact *p* < 0.01, adjusted *z* = -3.01) than those in the 3PP group (3.24 ± 0.47); s14, regarding the feeling of being dragged (*p* < 0.01, 2^∗^1-sided exact *p* < 0.01, adjusted *z* = -3.33; 1PP: -0.44 ± 0.10; 3PP: 0.05 ± 0.10); and s15, about the feeling of sliding (*p* < 0.01, 2^∗^1-sided exact *p* < 0.01, adjusted *z* = -2.70; 1PP: -0.17 ± 0.13; 3PP: 0.18 ± 0.10). Note that the raw data of s14 (about being dragged) and s15 (about sliding) seem to have the 1PP higher than the 3PP group, but after the ipsatization process, the pattern was inverted. S7 (about standing) and s9 (about sense of agency) did not differ between groups (Figure 6C). The retrospective power analysis resulted in power = 0.80, η^2^ = 0.72.

### Two-Dimensional Mood Scale

For pleasure, we found no effect of interaction or main effect of time, meaning that the pleasure level did not change before and after each session, so it did not affect the physiological recording. We found though a main effect of session [*F*(10, 300) = 12.31, *p* < 0.01], showing a decline in the level of pleasure, but independently of the group or before/after the session itself. Consequently, we averaged the results for pleasure for pre- and post-sessions, and we confirmed that there are no significant differences.

As for arousal, we found a comparable pattern: although there was no effect of interaction, there was a main effect of session [*F*(10, 300) = 28.27, *p* < 0.01], again showing a decline in the pleasure levels as the intervention continued. We found no differences in time or group, combining the pre and post results for all sessions. In summary, no changes in mood (pleasure and arousal specifically) were detected comparing the two groups before or after each session, so they unlikely affected the physiological recordings.

### Stroop Task’s RT and ER

Regarding the RT measurements, in 2 × 2 ANOVA to compare the data recorded during the baseline assessment, the interaction between factors [*F*(1, 40) = 6.13, *p* = 0.02] and also the main factor condition [*F*(1, 40) = 95.31, *p* < 0.01] were significant, with RT in the neutral condition (1,548.22 ± 45.96 ms) inferior to that in the incongruent condition (1,933.42 ± 52.25 ms). The main factor group was not significant. Therefore, we can conclude that, independently of group assignment, on average, all subjects showed the typical Stroop interference, before beginning the RCT.

**FIGURE 7:**
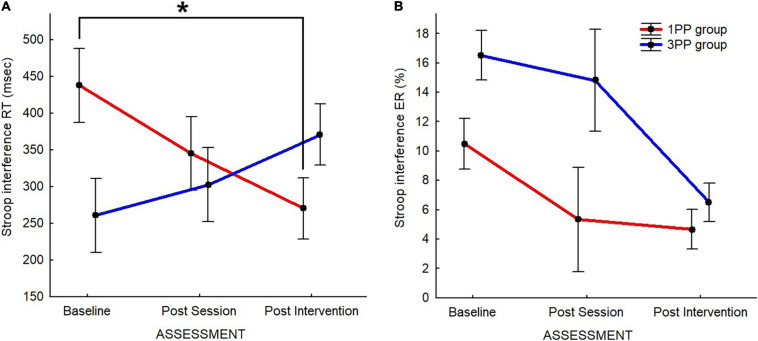
Line plots of the Stroop interference (incongruent–neutral condition) results for the Stroop task’s response time (RT) **(A)** and error rate (ER) **(B)**. For both, *red line* represents the results of the first-person perspective (1PP) group and *blue line* the results of the third-person perspective (3PP) group. Stroop interference results for response time (in milliseconds) **(A)** and error rate (in percent of error) **(B)** across the three assessment time points: baseline, post-session, and post-intervention. Significant difference (*p* < 0.05) is highlighted with *asterisk*. *Vertical black bars* denote plus/minus standard errors.

In the 2 × 3 ANOVA to compare the Stroop interference between groups and time of assessment, we found that the effect of interaction was significant [*F*(2, 80) = 5.24, *p* < 0.01]. At *post hoc* comparison, the Stroop interference in the 1PP group during the post-intervention assessment (270.20 ± 41.78) was significantly lower (*p* = 0.01) than that at baseline (437.80 ± 50.58), but not different with respect to the post-session assessment (345.07 ± 50.36). Although they clearly have opposite patterns (Figure 7A), it is worth noticing that there were no differences between groups at the assessments, except at baselines (*p* = 0.02; 1PP: 437.80 ± 50.58; 3PP: 260.59 ± 50.58). In the 3PP group, no differences were detected. The retrospective power analysis resulted in power = 0.80, d*z* = 0.25.

Regarding the ER measurements, in the Wilcoxon test to compare the data collected during the baseline assessment, the comparison between neutral (1.82 ± 0.39) and incongruent (15.79 ± 1.41) resulted significant (*p* < 0.01, *z* = 5.44), with the incongruent higher than the neutral, therefore showing the typical Stroop interference also for the ER. If we eventually compare between groups with the Mann–Whitney *U* test, we confirm that there was no difference.

In the Mann–Whitney *U* test to compare between groups and time of assessment, ER (incongruent–neutral) was significantly different (*p* = 0.04, 2^∗^1-sided exact *p* = 0.04, adjusted *z* = −2.06) between groups at baseline (1PP: 12.17 ± 1.71; 3PP: 16.51 ± 1.80), but not at post-session or at post-intervention (Figure 7B).

### fNIRS Data

While we did not find any significant difference in HHb, we found a significant effect of group × condition interaction [*F*(2, 64) = 3.32, *p* = 0.04] for O_2_Hb in the 2 × 3 ANOVA. At *post hoc* (Duncan’s test), the only significant difference was between post-intervention comparing groups (*p* = 0.01), showing an increased activation in the 1PP (0.14 ± 0.08) with respect to the 3PP (−0.09 ± 0.08) group (Figure 4B). The retrospective power analysis resulted in power = 0.90, d*z* = 0.40.

There was no difference in activation comparing the baselines of the two groups. Different with respect to predictions and the previous results, we did not find any difference in post-session activation but, as mentioned before, only at post-training assessment. This result seems to be coherent with the Stroop task results of RT. Interestingly, plotting the O_2_Hb results by trial (showing the timing of activation with respect to the Stroop task stimulus), there was clearly an increased activity in post-intervention, 6–8 s after displaying the visual stimulus (Figure 4C).

### Correlations

In Spearman’s correlation (to test for correlations between measurements during the vHIE), we found that the HR during the fast walking phase positively correlated with s1 [*r* = 0.42, *t*(*N* - 2) = 2.94, *p* = 0.01, FDR-adjusted *p* = 0.01], s3 [*r* = 0.41, *t*(*N* - 2) = 2.85, *p* = 0.01, FDR adjusted *p* = 0.01], and s4 [*r* = 0.49, *t*(*N* - 2) = 3.55, *p* < 0.01, FDR-adjusted *p* < 0.01]. A negative correlation was found for s2 [*r* = -0.43, *t*(*N* - 2) = -3.01, *p* < 0.01, FDR-adjusted *p* = 0.01]. We found no significant correlations between HR and the subjective ratings in the slow walking phase or between the HR and the questionnaire during the static phase.

Concerning the correlation between HR and the offline questionnaire’s results, we found positive significant correlations between HR during the fast walking phase and s5 [*r* = 0.38, *t*(*N* - 2) = 2.66, *p* = 0.01, FDR-adjusted *p* = 0.02], s6 [*r* = 0.44, *t*(*N* - 2) = 3.10, *p* < 0.01, FDR-adjusted *p* = 0.01], s7 [*r* = 0.38, *t*(*N* - 2) = 2.61, *p* = 0.01, FDR-adjusted *p* = 0.01], s8 [*r* = 0.39, *t*(*N* - 2) = 2.70, *p* = 0.01, FDR-adjusted *p* = 0.02], s11 [*r* = 0.42, *t*(*N* - 2) = 2.96, *p* = 0.01, FDR-adjusted *p* = 0.01], s12 [*r* = 0.44, *t*(*N* - 2) = 3.09, *p* < 0.01, FDR-adjusted *p* = 0.01], s13 [*r* = 0.47, *t*(*N* - 2) = 3.40, *p* < 0.01, FDR-adjusted *p* = 0.01], s14 [*r* = 0.38, *t*(*N* - 2) = 2.63, *p* = 0.01, FDR-adjusted *p* = 0.01], and s15 [*r* = 0.38, *t*(*N* - 2) = 2.58, *p* = 0.01, FDR-adjusted *p* = 0.01]. s9 and s10 did not correlate with HR, and we found no correlations between statements and the HR during the slow walking phase, as it happened for the online questionnaire.

In Pearson’s correlation (to test for correlations between measurements during the assessment), we found a significant negative correlation between RT Stroop interference and O_2_Hb signal during the baseline assessment (*r* = -0.39, *p* = 0.02, FDR-adjusted *p* = 0.04) and a significant positive correlation during the post-session assessment (*r* = 0.37, *p* = 0.03, FDR-adjusted *p* = 0.03), but we did not find significant correlations during the post-intervention assessment. As for ER, we did not find correlations with the O_2_Hb data.

In Spearman’s correlation (to test for correlations between measurements during the vHIE and measurements during the assessment), after the FDR adjustment, only the correlations between s9 and the O_2_Hb signal in the post-intervention assessment resulted significant [*r* = 0.31, *t*(*N* - 2) = 2.03, *p* = 0.04, FDR-adjusted *p* = 0.04].

## Discussion

The present study not only aimed at confirming previous results about the cognitive benefits of virtual training (Burin et al., 2020) but also intended to provide new knowledge concerning the long-term effects of this intervention on the elderly population. Here, we proposed a vHIE intervention, with similar characteristics to that in Burin et al. (2020) and Burin et al. (2019c): with the participants sitting still, the virtual body alternated sequences of fast and slow walking while we measured the heart rate and questionnaires during the virtual training and Stroop task and the cortical activity at different time points during the intervention. The main differences with respect to our previous study are the target sample and the timing of the intervention: in this case, we recruited a sample of over 60-year-old participants (organized into two groups, 1PP and 3PP, according to the visual perspective of the virtual body), and we modified the intervention itself in order to repeat the vHIE for 6 weeks, twice a week, for 20 min each session (instead of the 8 min for one session in the previous study). Coherently, we repeated the cognitive assessments before the beginning of the intervention, right after the first session, and at the end of the entire intervention. We will discuss the present findings starting from the initial hypothesis made in Section “Introduction.”

### Findings on Body Ownership, Agency, and Physiological Effect During the vHIE (Hypothesis 1) Are Confirmed in the Elderly Across the Long-Term Intervention

In the present study, we confirm, thanks to the questionnaires’ results, that the full-body illusion can cause a sense of body ownership over the virtual body, but only when the avatar is displayed coherently with the participants’ perspective (i.e., in the 1PP). Here, we confirm that the visual perspective seems to be the necessary condition for the ownership illusion to arise (Kokkinara et al., 2016; Burin et al., 2020; Neyret et al., 2020), while other stimulations, such as tactile, might contribute to increase it (Maselli and Slater, 2013), but are not as determinant as the visual channel.

We also confirm here that, despite the discrepancy between the real (no actions) and virtual (fast and slow walking) actions, the sense of agency over the virtual movements is, to some extent, preserved. The possibility to transfer the sense of agency to an agent other than our own body is not entirely a novelty: revisited versions of the rubber hand illusion involving movements proved how the sense of ownership over a fake hand (induced by visuotactile stimulation) can be so consistent to persist if it moves, inducing the sense of agency over the embodied movement (Kalckert and Ehrsson, 2012, Kalckert and Ehrsson, 2014; Burin et al., 2017). The full-body illusion replicates and strengthens this result by involving the entire body and allowing the overlap of the real and the physical body (Ehrsson, 2007; Slater, 2018), thanks to technologies such as IVR. In this context, while the sense of ownership is maintained, it is possible to manipulate several aspects related to the sense of agency. For example, the same illusion is effective even when there is a discrepancy between the intended and the executed movements: within certain spatial or temporal constrains (Burin et al., 2019a), the seen/executed movements can differ from the intended ones, without disrupting the sense of agency or even increasing it (Aoyagi et al., 2019).

Different with respect to the previous studies, here, the real person’s body is still and no instructions were given about any movements (Kokkinara et al., 2016; Burin et al., 2020), so there is no motor intention created. Therefore, there is no direct contrast in the neurocognitive comparator model, which compares the predicted action and the executed one (Frith et al., 2000). It is worth noticing that for the 1PP group, the recorded answers to the questionnaire’s statements related to the sense of agency are, on average, between 4/7 (“I don’t know”) and 5/7 (“I slightly agree”), but still higher than those of the 3PP group or with respect to the control statements: possibly, in a situation of uncertainty, where the virtual body in the 1PP is considered as one’s own (see answers to sense of body ownership statements in Tables 1, 2) and it is moving, people tend to attribute the agency of the seen movements to themselves, driven by the increased sense of ownership over the avatar (Burin et al., 2017, 2020). The absence of a motor plan might have positively contributed to this phenomenon because the seen movements (performed by one’s own virtual body) do not directly contrast the plan itself, but they can somehow integrate and be justified by the increased sense of body ownership (“this is my body–my body is moving–I am moving”). In this case, what happens might be an *a posteriori* reconstruction of the motor intention, based on the sense of body ownership, while normally it is a forward process, starting with the intention itself.

These levels of sense of subjective body ownership and agency might be sufficient to determine consequent reactions on a physiological level, i.e., increasing the heart rate coherently with the virtual movements, despite the participant being completely still. Even though this phenomenon has been observed in previous studies (Moseley et al., 2008; Kokkinara et al., 2016; Matamala-Gomez et al., 2020), here, the virtual movements alternate sequences of fast and slow walking (2 min each) instead of having a constant animation at the same speed with a final rush, which makes the physiological reaction even more reliable. The correlations between the heart rate results during the fast walking phase and the sense of body ownership and agency during the vHIE indicate that the more one feels the virtual body and the virtual movements as one’s own, the more the heart rate increases when the avatar is fast walking, confirming the link between the subjective experience of the illusion and the physiological reaction.

It also proves, once again, that the IVR illusion is extremely effective: in fact, the physiological response to the HIE seems to be somehow comparable to what happens during the same training performed in real life (Kujach et al., 2017), even though the heart rate during the fast walking phase of the vHIE shows peaks much lower than its real version.

Curiously, ratings of the online and offline questionnaires seem to be quite similar in the sample of elderly in this study and that in our previous study with young participants (Burin et al., 2020). Despite some argued differences in the sense of body ownership (Riemer et al., 2019) or in the potential strength of the multisensory illusion in the two populations (Marotta et al., 2018; Serino et al., 2018; Hide et al., 2021), the virtual illusion is quite effective independently of age (Palomo et al., 2018). The same illusion in the elderly is constant across the long-term study since there are no differences between sessions of the intervention in both groups. Independently of the experimental procedures, there might be a certain level of individuality, personality traits, or undetected components that establish the singular adherence to the multisensory illusion (Marotta et al., 2016; Burin et al., 2019b).

### Previous Findings on Acute Cognitive and Neural Benefits of the vHIE (Hypothesis 2) Are Not Replicated in the Elderly

In the previous study with vHIE on young participants, the main outcome resulted from diminished response time at the Stroop task immediately after one session of virtual training in the 1PP condition (Burin et al., 2020). Here, right after the first session of vHIE in the 1PP group, we did not find the same effect replicated in the senior sample. This result is further set by the absence of an increased activation over the lDLPFC, as described by the fNIRS results. Nonetheless, we observed a clear trend from the baseline (before the beginning of the intervention) to the post-session assessment, where the response time at the Stroop task clearly decreased in the experimental group only.

This is the first attempt to compare the short- and long-term cognitive effects of a training performed virtually, to the best of our knowledge. While several studies have confirmed the potential effects of long-term interventions with different types of training, from exergames to brain trainings (Wollesen et al., 2020), little is known about the acute effects of virtual motor trainings on the elderly. A previous study with non-immersive virtual reality measuring different outcomes, including executive functions, went in a similar direction with respect to the current result: institutionalized elderly people did not show an improvement after a single session of exergames (Monteiro-Junior et al., 2017b).

Our previous study’s only direct comparison is where young people showed an acute improvement in cognitive functions right after 8 min of vHIE in the 1PP condition. Perhaps, the crucial difference here is the population itself: it is quite established that body representations are updated less quickly and efficiently with age because of the deteriorated sensory modalities (Poliakoff et al., 2006; Kuehn et al., 2018), showing, for example, a bias in proprioceptive judgment (Riemer et al., 2019), which may cause falls and reduced manual dexterity. Even in the context of multisensory illusions, they seem to have difficulties in merging sensory cues coming from different sources (Hay et al., 1996; Graham et al., 2014; Kállai et al., 2017). Someone argued that adolescents and young adults need to have a more flexible self-perception with respect to the elderly since their appearance change more quickly (Tajadura-Jiménez et al., 2012), while someone else supported the associative learning theory based on the fact that the elderly have a longer experience of spatiotemporal matching sensory experiences, which in turn makes the probability of an uncertain situation, such as an experimentally induced multisensory illusion, *a priori* less likely to happen (Armel and Ramachandran, 2003; de Vignemont, 2010). Despite these possible explanations, the rubber hand illusion and other variations can be effectively induced in the elderly, as we have shown in this study, with underlying processes comparable to those in the younger population. It has been demonstrated that older adults perceive their own hand as closer to their own body with respect to younger adults before any kind of visuotactile stimulation; however, this proprioceptive bias does not correlate with the behavioral (i.e., the proprioceptive drift) and subjective measurements (i.e., questionnaire), but the strength of the illusion is persistent in a comparable manner to young people (Riemer et al., 2019). For these reasons, it might be possible that the full-body illusion (despite being present and persistent), but especially its consequences on cognitive and neural functions, requires more time to adapt in such an established representation of body and movements, while young adults are more prone to modifications of their relatively flexible system. Considering we did not perform assessments other than the post-session and post-intervention, we cannot argue here about the exact timing when the cognitive performance actually improves, i.e., which is the minimum amount of time necessary for a training, such as this one, to be effective on a cognitive and neural level. Also, since we did not run a follow-up measurement, we cannot argue about the stability of this effect. This might be an issue to be further studied.

### Increased Cognitive and Neural Improvement After the Entire Intervention (Hypothesis 3) Are Reported as Long-Term Effects of the vHIE Performed by One’s Own Virtual Body

As the main outcome of the study, we found a decreased response time at the Stroop task in the assessment after the 6-week intervention with respect to the baseline in the 1PP group only. This result is doubly confirmed by the fNIRS results, where an increased activity over the lDLPFC (generally considered as part of the underlying neural network of the Stroop task) was detected, again only after the entire intervention, in the 1PP group. Interestingly, the two results significantly correlate, i.e., a shorter response time in the Stroop task corresponds to an increased neural activation on the lDLPFC. The strength of the illusion (in terms of sense of body ownership and agency over the moving virtual body in 1PP) and its repetitions across the intervention triggered the alterations in the body and movement representation of the elderly participants, necessary to induce first the physiological reaction and then the high-level cognitive response, supported by the neural activation. As argued in Burin et al. (2020), we believe that the chain of events that culminates with the improved behavioral output starts with the manipulation of sense of body ownership and agency, thanks to the virtual illusion (Slater, 2009), which has been proven to be very effective in several previous studies on a subjective (Maselli et al., 2016), motor (Burin et al., 2019a), physiological (Kokkinara et al., 2016; Martens et al., 2019; Matamala-Gomez et al., 2020), or even social (Maister et al., 2015; Bedder et al., 2019) level. In this case, we focused the effectiveness of the illusion on the subjective and physiological responses as we found especially higher ratings to the online questionnaire during the vHIE training in the 1PP, but also an increased heart rate while the virtual body in the 1PP was fast walking (also, the two measurements correlate, i.e., the increased heart rate corresponds to higher ratings to s1 and s3, statements for ownership and agency, respectively). These two data, combined in consideration of all sessions, confirm the success of the illusion itself and constitute the first and necessary “building block” for the illusion to arise (Maselli and Slater, 2013) and to determine its consequent effects. Comparable to what happens after a real physical activity (Byun et al., 2014; Kujach et al., 2017), here, we crucially found an improvement in the cognitive task, specifically at the response time.

From a neurobiological perspective, it has been described that intermittent acute physical exercises, with various intensities, trigger the release of noradrenaline, dopamine, and acetylcholine from the nuclei, possibly triggered, in turn, by the increased general physiological activation of the organism (e.g., heart rate) (Lambourne and Tomporowski, 2010; Dietrich and Audiffren, 2011). These nuclei are structurally and functionally connected to the hippocampus and prefrontal cortex (Arnsten, 2011). The latter is critically involved in executive functions and specifically to those involved in the Stroop task (MacDonald et al., 2000) and related to physical activity (Yanagisawa et al., 2010; Byun et al., 2014; Kujach et al., 2017). Here, we confirm these results by showing an increased activation with a coincident timeline with respect to the task itself (i.e., in Figure 6, there clearly is a peak at 6–8 s after the stimulus onset, proving its connection with the task). Inhibitory control, which is one of the key functions in the execution of the Stroop task, seems to be critical to selecting or discarding unrelated information that can interfere with the completion of a specific goal, and it seems to decline with age (Friedman and Miyake, 2004). Although, a recent study proved how overall inhibitory control at the Stroop task can be improved after an acute training even in aged people (Fujihara et al., 2021). Because of the behavioral and neural results of the present study, this neurobiological explanation might be a potential interpretation; unfortunately, as it happened in the previous study with young subjects, we did not find any significant differences in the TDMS, and specifically in the arousal subscale. This might be explained by the overall heart rate activation, which was significantly higher during the running phase, but never really high, and surely not as it happens with real physical exercise. It is possible that the virtual illusion created the conditions for the physiological activation, but that activation was not enough to be reflected in a subjective increase of the arousal level. Even though the ratio of the heart rate increase during the 1PP training is way lower with respect to what happens in real physical activity, we assume that the subjective illusion and its physiological activation on the real person’s body was enough to determine the cognitive outcome, which was sustained and mediated by the neural activation over the lDLPFC. As previously argued, it also seems that the repetition of the training was fundamental for the elderly since we did not find the same effect in the acute but only in the long-term intervention.

In summary, we believe that comparable processes happen after a “real” physical exercise and after a virtual one, except that, in the latter, the actual execution of movements is replaced by the virtual body: the illusion is so strong that the general bodily arousal (even though it is not subjectively perceived) might be enough to determine the release of neurotransmitters, comparable to what happens after a training with one’s own body. As explained in the first subsection of Section “Discussion,” we consider the sense of body ownership and agency over the avatar in the 1PP themselves. Their consequences on the real body are a crucial key component to connecting the perceptual level of the virtual illusion with the higher functions. In contrast with previous studies (Kujach et al., 2017), we cannot argue that the generic arousal activation supplies to that role because we did not find any significance of the TDMS. Other studies with combinations of exergames (such as the Kinect) and physical exercise, in some cases on the elderly with mild cognitive impairments, argue that the improved neural efficiency acts as an intermediate between the improvement of bodily and global cognitive functions (Ansai et al., 2017; Bacha et al., 2018; Morita et al., 2018; Liao et al., 2019, 2020; Wollesen et al., 2020), which might be in line with our results. Nevertheless, they clearly exploit “real” physical activity, actually performed by the physical body, or they test VR games acting specifically on certain cognitive functions, perhaps in combination with physical exercises: the novelty of our study lies in the manipulation of the sense of body ownership and agency over the virtual body, which is the only agent performing the training, while the real body is still. Despite this contradiction, the illusory feelings toward the avatar show consequences on different and higher functions, such as the cognitive one, after the long-term intervention.

### Limitations

We mentioned that the main outcome of this study is the response time of the Stroop task, although the other behavioral component is represented by the accuracy (i.e., percentage of error). We did not find any significant difference between assessments for the latter, but only a difference in the baseline between groups. It shows a pattern similar to the response time, decreasing after post-session and post-intervention assessments, but not significantly. This is consistent with the results shown by young participants (Burin et al., 2020), where it was possibly due to a contingency or a repetition effect (Hazeltine and Mordkoff, 2014), which seems to be specific for the error rate and not for speed.

A similar issue was with regard to the baseline assessments: for the error rate, as for the response time, we found a difference between groups, meaning that the two groups do not start from the same baseline level concerning the Stroop task. Possibly for this same reason, we did not find a significant difference in RT in the post-intervention assessment between groups, but a significant interaction and a significant difference in the 1PP group with respect to its baseline. Nonetheless, they show opposite patterns, and the main outcome of the response time correlates with the fNIRS data, the other measurement assessed during the Stroop task. Although the group allocation was done before the baseline assessment, it is possible that the selected participants started from different levels of speed.

The scores concerning the sense of agency statements in both online and offline questionnaires are between 3 and 4/7, around the middle point of the Likert scale, meaning uncertainty. In the online questionnaire, there is still a significant difference between groups (with 1PP higher than 3PP), but in the offline questionnaire, at s9, there is no difference between groups. These results are quite comparable to those in younger participants. As previously argued, it might be possible that this feeling of uncertainty about the explicit sense of agency is enough to attribute to oneself the seen movements, or at least show their physiological counterpart. It would be possible to further increase the sense of agency over the moving virtual body by including additional stimulations, such as vibrotactile feedbacks in correspondence to the walking–fast walking animation.

## Data Availability Statement

The raw data supporting the conclusions of this article will be made available by the authors, without undue reservation.

## Ethics Statement

The studies involving human participants were reviewed and approved by Ethics Committee of the Tohoku University Graduate School of Medicine. The patients/participants provided their written informed consent to participate in this study. Written informed consent was obtained from the individual(s) for the publication of any potentially identifiable images or data included in this article.

## Author Contributions

DB and RK conceptualized the study. DB developed and RK approved the methodology. DB implemented the software, organized the investigation and the database, performed the formal analysis, and provided the financial resources. RK was responsible for supervision. DB wrote the first draft of the manuscript. DB and RK edited and reviewed the manuscript. All authors contributed to the manuscript revision, read, and approved the submitted version.

## Conflict of Interest

The authors declare that the research was conducted in the absence of any commercial or financial relationships that could be construed as a potential conflict of interest.

## Footnotes

1 https://www.oculus.com/

## Abbreviations

RHI: rubber hand illusion
IVR: immersive virtual reality
vHIE: virtual high-intensity intermittent exercise
1PP: first-person perspective
3PP: third-person perspective
lDLPFC: left dorsolateral prefrontal cortex
HR: heart rate
TDMS: Two-Dimensional Mood Scale
fNIRS: functional near-infrared spectroscopy
O_2_Hb: oxygenated hemoglobin
HHb: deoxygenated hemoglobin
RT: response time
ER: error rate.
