# Supplementary material for: Repeated Exposure to Illusory Sense of Body Ownership and Agency Over a Moving Virtual Body Improves Executive Functioning and Increases Prefrontal Cortex Activity in the Elderly
Source: Front Hum Neurosci. 2021 May 31;15:674326. doi: 10.3389/fnhum.2021.674326 (PMC8200494; doi:10.3389/fnhum.2021.674326)
Supplement: Supplementary file 1 [file Data_Sheet_1.DOCX]

Burin D., Kawashima R. *Repeated exposure to illusory sense of body ownership and agency over a moving virtual body improves executive functioning and increases prefrontal cortex activity in the elderly.*

**Supplementary material.**

**Table S1.** Results of comparisons (Friedman Anova) between sessions of each statement individually of the online questionnaire on sense of body ownership and agency during the static phase of the vHIE. The first column represents the statement of the questionnaire (from s1 to s4) and the second column reports the p values (marked * if significant) comparing between sessions. If a comparison was detected as significant, post-hoc details are reported (ipsatized data±SE).

| statement | p* value |
| --- | --- |
| s1 | .16 |
| s2 | .19 |
| s3 | .22 |
| s4 | .01* |

*in session 9 (-0.01±0.32) and session 10 (-0.02±0.29) s4 (control statement about the sense of agency) is significantly lower than the other sessions.

**Table S2.** Results of comparisons (Friedman Anova) between sessions of each statement individually of the online questionnaire on sense of body ownership and agency during the training phase of the vHIE. The first column represents the statement of the questionnaire (from s1 to s4), the second column represents the minute of the vHIE when the statement was administered, and the third column reports the p values (marked * if significant) comparing between sessions.

| statement | minute | p* value |
| --- | --- | --- |
| s1 | 3 | .96 |
|  | 8 | .38 |
|  | 13 | .85 |
|  | 18 | .28 |
| s2 | 3 | .65 |
|  | 8 | .42 |
|  | 13 | .23 |
|  | 18 | .12 |
| s3 | 3 | .72 |
|  | 8 | .93 |
|  | 13 | .82 |
|  | 18 | .39 |
| s4 | 3 | .19 |
|  | 8 | .22 |
|  | 13 | .35 |
|  | 18 | .10 |

**Table S3.** Results of comparisons (Friedman Anova) between repetitions of the same statement, within sessions, of each statement individually of the online questionnaire on sense of body ownership and agency during the training phase of the vHIE. The first column represents the statement of the questionnaire (from s1 to s4), the following columns represent the sessions; for each comparison, the p values (marked * if significant) are reported. If a comparison was detected as significant, post-hoc details are reported (ipsatized data ±SE).

| statement | session | | | | | | | | | | |
| --- | --- | --- | --- | --- | --- | --- | --- | --- | --- | --- | --- |
|  | 1 | 2 | 3 | 4 | 5 | 6 | 7 | 8 | 9 | 10 | 11 |
| s1 | .81 | .73 | .58 | .16 | .27 | .07 | .94 | .30 | .31 | .58 | .72 |
| s2 | .44 | .42 | .30 | .54 | .70 | .12 | .78 | .31 | .22 | .28 | .89 |
| s3 | .33 | .88 | .80 | .51 | .19 | .14 | .31 | .57 | .43 | .87 | .22 |
| s4 | .13 | .44 | .50 | .20 | .35 | .06 | .02* | .18 | .70 | .64 | .76 |

*at 8 min (0.61±0.23) is sign higher than 3 min (.35±0.20), 13 min (0.42±0.21) and 18 min (0.42±0.22).

**Table S4.** Results of comparisons (Friedman Anova) between sessions of each statement individually of the offline questionnaire on sense of body ownership and agency during the training phase of the vHIE. The first column represents the statement of the questionnaire (from s5 to s15) and the second column reports the p values (marked * if significant) comparing between sessions. If a comparison was detected as significant, post-hoc details are reported (ipsatized data ±SE).

| statement | p* value |
| --- | --- |
| s5 | .70 |
| s6 | .29 |
| s7 | .11 |
| s8 | .30 |
| s9 | .58 |
| s10 | .15 |
| s11 | .76 |
| s12 | .73 |
| s13 | .62 |
| s14 | .34 |
| s15 | .52 |
